# Sleep apnea-COPD overlap syndrome is associated with larger left carotid atherosclerotic plaques

**DOI:** 10.3389/fcvm.2023.1104377

**Published:** 2023-03-21

**Authors:** Pedro Landete, Carlos Ernesto Fernández-García, José M. Muñoz, Alfonsi Friera, Julio Ancochea, Águeda González-Rodríguez, Carmelo García-Monzón

**Affiliations:** ^1^ Servicio de Neumología, Hospital Universitario de La Princesa, Instituto de Investigación Sanitaria del Hospital Universitario de La Princesa, Madrid, Spain; ^2^ Facultad de Medicina, Universidad Autónoma de Madrid, Madrid, Spain; ^3^ Liver Research Unit, Unidad de Investigación, Hospital Universitario Santa Cristina, Instituto de Investigación Sanitaria del Hospital Universitario de La Princesa, Madrid, Spain; ^4^ Servicio de Radiodiagnóstico Hospital Universitario de La Princesa, Instituto de Investigación Sanitaria del Hospital Universitario de La Princesa, Madrid, Spain; ^5^ Instituto de Investigaciones Biomédicas Alberto Sols (Centro Mixto CSIC-UAM), Madrid, Spain; ^6^ Centro de Investigación Biomédica en Red de Diabetes y Enfermedades Metabólicas Asociadas (CIBERDEM), Madrid, Spain

**Keywords:** atherosclerosis, chronic obstructive pulmonary disease, carotid atherosclerosis, apnea, sleep obstructive apnea, overlap syndrome

## Abstract

**Background:**

Little is known about whether the overlap syndrome (OS) combining features of chronic obstructive pulmonary disease (COPD) and sleep apnea-hypopnea syndrome increases the risk of stroke associated with COPD itself.

**Methods:**

We prospectively studied 74 COPD patients and 32 subjects without lung disease. Spirometry and cardiorespiratory polygraphy were used to assess the pulmonary function of the study population and ultrasound measurements of intima media thickness (IMT) as well as the volume of plaques in both carotid arteries were also evaluated.

**Results:**

Polygraphic criteria of OS were met in 51% of COPD patients. We found that 79% of patients with OS and 50% of COPD patients without OS had atherosclerotic plaques in the left carotid artery (*p* = 0.0509). Interestingly, the mean volume of atherosclerotic plaques was significantly higher in the left carotid artery of COPD patients with OS (0.07 ± 0.02 ml) than in those without OS (0.04 ± 0.02 ml, *p* = 0.0305). However, regardless of the presence of OS, no significant differences were observed in both presence and volume of atherosclerotic plaques in the right carotid artery of COPD patients. Adjusted-multivariate linear regression revealed age, current smoking and the apnea/hypopnea index (OR = 4.54, *p* = 0.012) as independent predictors of left carotid atherosclerotic plaques in COPD patients.

**Conclusions:**

This study suggests that the presence of OS in COPD patients is associated with larger left carotid atherosclerotic plaques, indicating that OS might be screened in all COPD patients to identify those with higher risk of stroke.

## Introduction

Chronic obstructive pulmonary disease (COPD) is a chronic respiratory disease with a high global prevalence which is estimated to be currently the third leading cause of death worldwide and the numbers are rising (1, 2). Since COPD is a non-curable disease, smoking cessation is the only effective measure to prevent and slow its progression (3). The main endpoints in the COPD therapy are: to attenuate disease symptoms, to reduce the frequency and severity of exacerbations, and to improve the prognosis. Noteworthy, a new chronic respiratory disease combining features of COPD and sleep apnea-hypopnea syndrome, named overlap syndrome (OS), has been described. Although little is known nowadays about its clinical and prognostic impact, there is a growing evidence that the clinical outcomes of patients with OS may be more deleterious than those of patients with either COPD or sleep apnea alone (4, 5). The prevalence of OS in the general population has been assessed in distinct epidemiological studies with estimations varying from 10% to 66% (6, 7).

COPD is associated with a high morbidity and mortality largely due to the presence of metabolic and cardiovascular co-morbidities, such as obesity, type 2 diabetes, non-alcoholic fatty liver disease and a wide spectrum of atherosclerosis-related cardiovascular disorders (CVD) (8). Regarding the latter, distinct meta-analysis of observational clinical studies showed that COPD patients have 2-fold higher risk of CVD than subjects without COPD, being ischemic heart disease, ischemic stroke and peripheral artery disease the most frequently observed CVD in COPD patients (9, 10). In fact, several studies have investigated whether CVD are more prevalent in certain phenotypes of COPD patients without conclusive results so far. It has been observed that cardiovascular co-morbidities are not only limited to those with more advanced airflow obstruction, but they are indeed present across the wide spectrum of disease severity (11, 12). Regarding sleep apnea, it has been described distinct inflammatory factors which might cause the progression of atherosclerosis, thus increasing the risk of cardiovascular and cerebrovascular diseases (13). In this context, Trzepizur et al. have recently reported that patients with obstructive sleep apnea and elevated hypoxic burden are at higher risk of a cardiovascular event and all-cause mortality (14). Interestingly, OS has been linked to higher cardiovascular morbidity, poorer quality of life, and higher frequency of COPD exacerbations (15), but these findings need to be confirmed in further clinical studies.

Atherosclerosis is the underlying cause of CVD in most cases (16) and it is considered the first cause of death in CVD as well as a major cause of total deaths (17). Atherosclerosis is a chronic and systemic disease characterized by promoting cholesterol influx in the vascular wall, leading to fatty streaks and fibrotic streaks in early stages, and prompting generation of a necrotic core with thrombogenic capacity in advanced complicated plaques (18). Currently, it is quite unpredictable to know who is going to suffer from atherothrombosis, which depends on the vulnerability of the plaque. Noteworthy, most of the patients are asymptomatic, especially in early stages. For these reasons, the current approaches to improve the diagnosis and prognosis are non-invasive imaging techniques to better characterize vessel morphology as well as biomarkers discovery. Carotid atherosclerosis (CAS) plays a fundamental part in the occurrence of ischaemic stroke, and some morphological characteristics like plaque volume are promising as imaging biomarkers of carotid plaque vulnerability (19). Moreover, mechanisms underlying the pathogenic link between sleep apnea and carotid atherosclerosis may be different for carotid plaque development than for the increase of carotid intima-media thickness (20). On the other hand, despite the growing appreciation of the importance of atherosclerosis in COPD patients, there is still considerable ambiguity about its prevalence and clinical impact. Therefore, in this prospective cross-sectional study we wanted to explore the prevalence of and risk factors for CAS among patients with COPD, either with or without OS, in order to determine whether the coexistence of the OS in COPD patients might impact on the development of CAS.

## Patients and methods

### Study population

This study was performed in agreement with the Declaration of Helsinki, and with local and national laws. The Institution's Clinical Research Ethics Committee approved the study procedures (report reference, PI16/2,800), and all participants signed an informed written consent before inclusion in the study, providing permission for their medical data to be anonymously used for research.

This prospective cross-sectional study included consecutive patients with clinical, spirometric and polygraphic criteria of COPD, with or without OS, among those who attended to the outpatient clinics of the Respiratory Service at Hospital Universitario de La Princesa (Madrid, Spain) during a 6 months period. In parallel, volunteers who had both spirometry and sleep polygraphy parameters within normality were included in the study and considered as control subjects with normal lung parameters (NLP). Patients and controls were excluded if they drank more than 20 g/day of alcohol, had a diagnosis of asthma or cancer or any concomitant severe clinical disorder. In addition, they were also excluded if had analytical evidence of iron overload, were seropositive for autoantibodies and/or for hepatitis B virus, hepatitis C virus, and human immunodeficiency virus as well as those having actively drugs such as cannabis and cocaine among others.

### Demographic, clinical and biochemical assessment

Clinical examination was performed to all participants in this study including a detailed interview with special emphasis on smoking pattern, alcohol and drugs abuse (cannabis and cocaine) and medications use, history of diabetes and arterial hypertension as well as measurements of weight and height. Body mass index (BMI) was calculated and obesity was defined as BMI ≥ 30 kg/m2. After overnight fast, venous blood samples of each participant were obtained to test serum levels of different biochemical and metabolic parameters. Insulin resistance was calculated by the homeostasis model assessment method (HOMA-IR) (21). Metabolic syndrome was defined according to the ATP III criteria (22).

### Spirometry

To assess the diagnosis and severity of COPD, spirometry was performed to all participants by using a JAEGER™ spirometer (Vyaire Medical, Madrid, Spain) which meets all the specifications required by the Spanish Respiratory Society, the European Respiratory Society and the American Thoracic Society. All patients and control subjects underwent pre- and post-bronchodilator spirometric determinations.

### Cardiorespiratory polygraphic study

All polygraphic studies were performed at night in the Sleep Laboratory of the Hospital Universitario de La Princesa by using validated procedures as previously described (23). Sleep studies were performed using a cardiorespiratory polygraphy (SOMNOscreenTM Plus, Randersacker, Germany), previously validated, with DOMINO analysis software (Domino Data Lab, San Francisco, CA). The interpretation of the register was carried out manually, although assisted by a computer, following the consensus recommendations for the diagnosis of apnea, hypopnea with desaturation of 3%, according to the recommendations for diagnosis of sleep apnea-hypopnea syndrome of the Spanish Society of Pneumology and Thoracic Surgery (24). Moreover, episodes of apnea were further characterized as central or obstructive as previously described (23). The presence of an apnea and hypopnea index (AHI) equal to or greater than 5 per hour of sleep was used as diagnostic criterion for certainty of sleep apnea. The severity of apnea-hypopnea was classified according to the value of AHI as mild (AHI, 5–14/h), moderate (AHI, 15–29/h) or severe (AHI ≥ 30/h). In addition, other pulmonary parameters were analyzed such as the oxygen desaturation index (ODI) and the percentage of sleep time with oxygen saturation below 90% (Tc90%). Both ODI and Tc90% were considered low when lower than 10 events/hour and 10%, respectively, and were considered high when equal or higher than 10 events/hour and equal or higher than 10%, respectively.

### Assessment of vascular damage

Distinct features of vascular damage were determined by using ultrasonography (Applio XG, Canon, Tokyo, Japan) to each patient as follows:

– Intima-media thickness (IMT) was measured in the distal 2 centimeters (cm) of both common carotid arteries. The methodology defined in the Mannheim consensus (ref tesis pedro 193) was used. Each participant had 3 carotid IMT (cIMT) measures by side, which were carried out and scored for quality by 2 experts vascular radiologists (AFR, JMO). We calculated cIMT for each participant as the average value of all measurements that met predefined quality standards.
– Volume of arterial plaques was determined following internationally accepted criteria (25). Arterial plaque was defined when 2 of the following criteria were met: 1- IMT > 1.5 mm. 2- Impression in the vascular lumen. 3- Abnormal wall texture. The plaque burden found in both carotid arteries (2 cm distal common carotid arteries and 1 cm distal internal carotid arteries) was calculated. This plaque load was expressed as the sum of the volumes of all plaques. All ultrasound measurements were performed by 2 experts vascular radiologists (AFR, JMO) using a 7 MHz linear probe (model PLT-704SB, Tokyo, Japan) and a high frequency volumetric linear probe model PLT-1204 MV probe (Canon, Tokyo, Japan) with a 3D/4D volumetric reconstruction software model Toshiba UIMV-A500A (Canon, Tokyo, Japan).

### Statistical analysis

The Kolmogorov–Smirnov test was applied to evaluate if variables were adjusted or not to a normal distribution. Qualitative variables are presented as absolute (number, n) and relative (percentage, %) frequencies. Quantitative variables are expressed as measures of central tendency (mean) and dispersion (standard deviation, SD). Qualitative data between groups were compared by Pearson's *χ*^2^ test or Fisher exact test as appropriate. The Student's *t* test was used to calculate the difference of the means in the variables that followed a normal distribution and the Mann–Whitney *U* test for the variables with a non-parametric distribution. Logistic regression analysis, adjusted by confounding variables (age, gender BMI, diabetes, arterial hypertension, total cholesterol, current smoking, post-FEV1 and number of exacerbations per year) was performed to identify independent polygraphic variables (AHI, ODI, and Tc90%) associated with the presence of atherosclerotic plaques in either the left or the right carotid artery in the study population. Multiple confounding factors of cardiovascular risk were evaluated, such as creatinine, glomerular filtration rate, albumin, glucose, insulin resistance assessed by HOMA-IR, triglycerides, low and high density lipoproteins, dyslipidemia, alkaline phosphatase and iron metabolism, as well as history of previous cardiovascular disease (atrial fibrillation, chronic heart failure, acute myocardial infarction, cerebrovascular disease and stroke) and medicament use (ACE inhibitors, angiotensin-II receptor antagonists and oral antidiabetics or insulin). Univariate and multivariate regression models were constructed, parameters were selected by likelihood ratio test, and Box-Tidwell procedure was used for testing linearity of logit. The goodness of fit was evaluated using the Hosmer–Lemeshow statistic. Significance was set at a value of p < 0.05. Statistical analysis was performed using SPSS software version 26.0 (SPSS Statistics, Armonk, NY: IBM Corp.).

## Results

### Characteristics of the study population

A total of 74 COPD patients and 32 subjects with NLP were included in the study according to inclusion and exclusion criteria. Demographic, anthropometric and analytical characteristics of the entire study population are detailed in Table 1. Overall, COPD patients were older and had insulin resistance, arterial hypertension and dyslipidemia more frequently than NLP subjects. Furthermore, COPD patients had significantly higher serum levels of ALT, GGT and ferritin than NLP controls. Regarding pulmonary function parameters, as expected, all spirometry parameters were significantly lower in COPD patients than in NLP subjects as well as basal and minimum oxygen saturation (Supplementary Table S1).

**Table 1:** Characteristics of the study population.

Features	NLP (*n* = 32)	COPD (*n* = 74)	p-value
Age (years)	54.38 ± 8.53	63.38 ± 6.57	<0.001
Gender			0.118
Women, *n* (%)	20 (62.5)	34 (45.9)	
Men, *n* (%)	12 (37.5)	40 (54.1)	
Body mass index (kg/m2)	28.91 ± 5.54	27.63 ± 5.80	0.208
Body mass index ≥30, *n* (%)	10 (31.3)	24 (32.4)	0.905
Glucose (mg/dl)	97.37 ± 13.37	101.84 ± 25.36	0.376
Insulin levels (µU/L)	11.54 ± 7.58	14.50 ± 9.65	0.131
HOMA-IR score	2.93 ± 2.27	3.94 ± 2.98	0.082
HOMA-IR score ≥2.5, *n* (%)	14 (43.8)	50 (67.6)	**0.021**
Glycated Hb (%)	5.63 ± 0.52	5.74 ± 0.58	0.108
Type 2 diabetes mellitus, *n* (%)	2 (6.3)	15 (20.3)	0.088
Hypertension, *n* (%)	9 (28.1)	41 (53.2)	**0.021**
Dyslipidemia, *n* (%)	7 (21.9)	36 (48.6)	**0.011**
Metabolic syndrome, *n* (%)	3 (9.4)	12 (16.2)	0.545
Triglycerides (mg/dl)	106.47 ± 66.57	125.03 ± 69.23	0.116
Total cholesterol	196.81 ± 39.17	198.23 ± 42.32	0.896
HDL-cholesterol (mg/dl)	57.53 ± 12.96	60.58 ± 22.21	0.422
LDL cholesterol (mg/dl)	115.22 ± 30.75	108.99 ± 38.66	0.404
ALT (IU/L)	18.78 ± 7.34	22.77 ± 9.09	0.033
AST (IU/L)	20.28 ± 5.61	23.18 ± 8.18	0.063
GGT (IU/L)	22.19 ± 13.03	36.55 ± 30.07	**0.003**
Iron (µg/dl)	78.47 ± 33.50	89.70 ± 29.33	0.124
Ferritin (ng/ml)	87.41 ± 72.14	134.26 ± 98.40	**0.021**
Transferrin (mg/dl)	244.26 ± 31.58	259.41 ± 39.47	0.051
Alkaline phosphatase (IU/L)	69.62 ± 22.69	71.23 ± 19.55	0.877
Lactate dehydrogenase (U/L)	182.72 ± 36.12	195.59 ± 36.12	0.140
Albumin (g/dl)	4.37 ± 0.27	4.38 ± 0.39	0.941
Platelets (109/L)	0.23 ± 0.07	0.24 ± 0.05	0.392
Total bilirubin (mg/dl)	0.57 ± 0.48	0.55 ± 0.29	0.467
C reactive protein (mg/L)	0.42 ± 0.59	0.42 ± 0.52	0.568

Data are shown as mean ± standard deviation or as number of cases (%). NLP, subjects with normal lung parameters; COPD, subjects with chronic obstructive pulmonary disease; HOMA-IR, homeostatic model assessment-insulin resistance; Hb, hemoglobin; HDL, high-density lipoprotein; LDL, low-density lipoprotein; VLDL, very low-density lipoprotein; ALT, alanine aminotransferase; AST, aspartate aminotransferase; GGT, gamma-glutamyltransferase.

### Prevalence of carotid atherosclerotic disease in the study population

**Figure 1:**
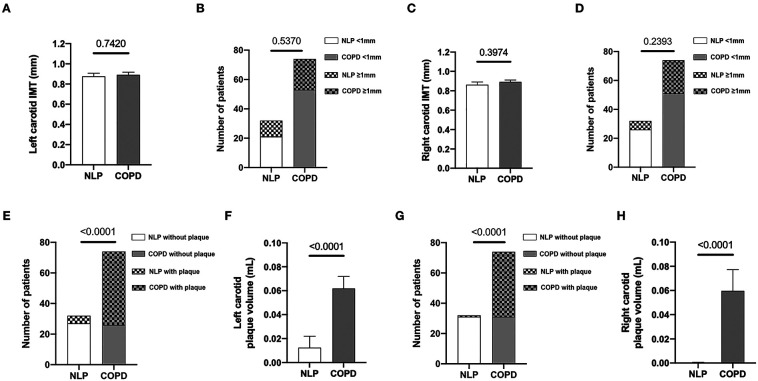
Prevalence of carotid atherosclerosis in the study population. Number of COPD patients and NLP subjects with left and right carotid IMT measurements (panels A and C, respectively) and differences between the study groups regarding mean IMT of left and right carotids (panels B and D, respectively). Number of COPD patients and NLP subjects with left and right carotid plaque measurements (panels E and G, respectively) and differences between the study groups regarding mean volume of left and right carotid plaques (panels F and H, respectively). COPD, chronic obstructive pulmonary disease, *n* = 74. NLP, normal lung parameters, *n* = 32.

To this end, left and right carotid artery were assessed by ultrasonography to measure IMT as well as to determine the number and volume of atherosclerotic plaques in the entire study population. We did not found differences in cIMT between COPD and NLP subjects (Figure 1, panels A–D) but atherosclerotic plaques, in both left and right carotids, were significantly more abundant and larger in COPD patients than in NLP subjects (p < 0.0001 for all cases) (Figure 1, panels E–H).

### Prevalence of overlap syndrome

We carried out cardiorespiratory polygraphic study to all COPD patients included in order to determine the prevalence of OS in our study cohort. According to internationally-accepted diagnostic criteria, COPD patients were stratified by the presence (AHI ≥ 5 events/hour) or absence of OS (AHI < 5 events/hour) and their baseline characteristics are shown in Supplementary Table S2. Overall, 38 out of 74 COPD patients (51%) had polygraphic criteria of OS, being the estimated prevalence of COPD-OS in our study cohort of 51%. These COPD-OS patients were predominantly men and had significantly higher serum GGT levels than those without OS. Pulmonary function parameters of COPD patients according to the absence or presence of OS are summarized in Table 2. To highlight, the majority of COPD patients with OS had a mild or moderate AHI (79%) and an ODI equal or higher than 10 (84.2%).

**Table 2:** Pulmonary function parameter in COPD population according to OS presence.

Features	Non-OS (*n* = 36)	OS (*n* = 38)	p-value
PRE FVC (ml)	2343.89 ± 830.86	2895.55 ± 983.88	**0.013**
PRE FVC (%)	70.48 ± 19.52	74.53 ± 20.19	0.407
PRE FEV (ml)	1200.08 ± 615.17	1634.47 ± 731.00	**0.008**
PRE FEV (%)	47.38 ± 21.18	55.57 ± 18.63	0.087
PRE FEV/FVC	49.95 ± 13.48	55.37 ± 12.35	0.082
POST FVC (ml)	2420.28 ± 831.29	2979.68 ± 1011.79	**0.009**
POST FVC (%)	71.65 ± 16.87	77.46 ± 20.12	0.159
POST FEV (ml)	1261,31 ± 659.57	1733.95 ± 769.66	**0.006**
POST FEV (%)	49.53 ± 22.46	59.42 ± 20.10	0.053
POST FEV/FVC	50.06 ± 13.66	56.60 ± 12.89	0.057
Basal saturation	92.81 ± 5.76	94.29 ± 2.57	0.337
Minimum saturation	78.92 ± 10.83	76.34 ± 8.57	0.067
Mallampati score	1.42 ± 1.00	1.47 ± 0.80	0.869
Dyspnoea grade	1.83 ± 1.03	1.74 ± 1.00	0.697
Physical activity scale	1.08 ± 0.81	1.16 ± 0.72	0.715
Exacerbations/year	0.83 ± 1.08	0.76 ± 1.15	0.718
COPD score (Gold)	1.61 ± 1.10	1.18 ± 1.01	0.087
AHI (events/hour)	1.67 ± 2.26	22.34 ± 17.14	**<0.001**
5–14, *n* (%)	0 (0)	15 (39.5)	
15–29, *n* (%)	0 (0)	15 (39.5)	**<0.001**
≥30, *n* (%)	0 (0)	8 (21.1)	
ODI (events/hour)	2.62 ± 2.65	25.58 ± 20.60	**<0.001**
≥10, *n* (%)	0 (0)	32 (84.2)	**<0.001**
Tc90%	35.19 ± 34.23	40.01 ± 33.63	0.245
≥10, *n* (%)	21 (58.3)	27 (71.1)	0.252

Data are shown as mean ± standard deviation or as number of cases (%). COPD, subjects with chronic obstructive pulmonary disease; OS, subjects with overlap syndrome; FVC, forced vital capacity; FEV, forced expiratory volume; AHI, apnea-hypopnea index; ODI, oxygen desaturation index; Tc90%, percentage of sleep time with oxygen saturation below 90%.

### Increased prevalence of left carotid atherosclerotic plaques in COPD patients with overlap syndrome

**Figure 2:**
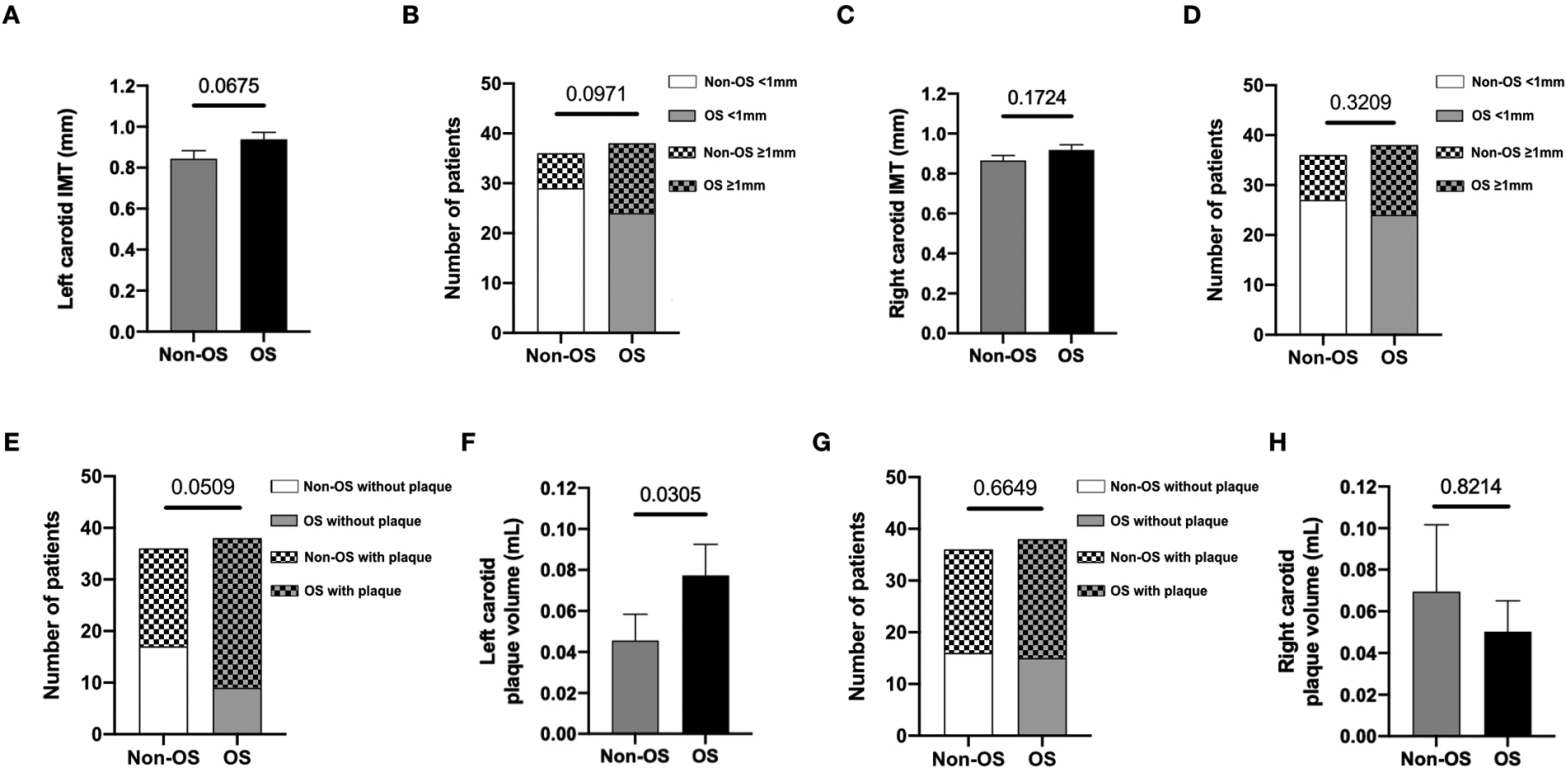
Increased prevalence of left carotid atherosclerosis in COPD patients with overlap syndrome. Number of COPD patients, with and without OS, with left and right carotid IMT measurements (panels **A** and C, respectively) and differences between the study groups regarding mean IMT of left and right carotids (panels B and D, respectively). Number of COPD patients, with and without OS, with left and right carotid plaque measurements (panels **E** and **G**, respectively) and differences between the study groups regarding mean volume of left and right carotid plaques (panels F and H, respectively). COPD, chronic obstructive pulmonary disease. Non-OS, non-overlap syndrome, *n* = 36. OS, overlap syndrome, *n* = 38.

No significant differences were observed in COPD patients with or without OS regarding cIMT measurements either in left or right carotid arteries (Figure 2, panels A–D), although there is a trend towards a slight increase in the left carotid artery, as well as in the number of patients with left cIMT above 1 mm, a widely used cut-off point as a surrogate marker of CAS. In the same line, we found that 30 out of 38 patients with OS (79%) and 18 out of 36 patients without OS (50%) had atherosclerotic plaques in the left carotid artery (p = 0.0509) (Figure 2, panel E). Interestingly, the mean volume of atherosclerotic plaques was significantly higher in the left carotid artery of patients with OS (0.07 ± 0.02 ml) than in those without OS (0.04 ± 0.02 ml, p = 0.0305) (Figure 2, panel F). Conversely, and regardless of the presence of OS, no significant differences were observed in both the presence and volume of atherosclerotic plaques in the right carotid artery of COPD patients with OS (Figure 2, panels G and H).

### Risk factors for left carotid atherosclerotic plaques in COPD patients

To further evaluate the impact of OS on the presence of left carotid atherosclerotic plaques in COPD patients, we performed multivariate logistic regression analysis in our study cohort to determine the associated risk factors. We performed univariate analysis of multiple traditional risk factors - detailed in Patients and Methods - for carotid atherosclerosis, COPD and sleep apnea, being the most relevant detailed in Supplementary Table S3. Multivariate logistic models were build with variables showing *p*-value <0.10 in univariate analysis, and revealed that only age [OR = 1.19 (95% CI: 1.07–1.32), p = 0.002], current smoking [OR = 10.50 (95% CI: 1.94–56.71), *p* = 0.006] and AHI ≥ 5 [OR = 4.54 (95% CI: 1.39–14.85) p = 0.012] significantly predicted an increased risk for left carotid atherosclerotic plaques in COPD patients (Supplementary Table S3).

## Discussion

The results of this study provides convincing evidence that COPD patients with concomitant polygraphic criteria of sleep apnea, which are defining the OS, had an increased mean volume of left carotid plaques than those without (0.07 ± 0.02 ml and 0.04 ± 0.02 ml, respectively, p = 0.0305), although the clinical relevance of this finding must be confirmed in further clinical studies. Besides, no differences were detected on right carotid arteries. In patients with OS, frequency of atherosclerotic plaques in the left carotid artery is increased compared to COPD patients without OS (79% and 50%, respectively, p = 0.0509) but the difference in number of patients is rather small and univariate analysis did not achieve statistical significance. For that reason, we investigated the influence of OS on the presence of left carotid plaques in the whole group of COPD patients (with and without OS) besides other traditional factors related to the progression of atherosclerosis, such as age, sex, obesity, diabetes, hypertension, hypercholesterolemia and active smoking. Even after adjustment for these potential confounding factors, the presence of OS in COPD patients was still significantly associated with the increased prevalence of left carotid plaques (OR, 4.54, p = 0.012). Among the lung function parameters that we used to assess the severity of OS, the frequency of AHI appeared to be more important that hypoxemia measured by ODI and Tc90%. On one hand, Tc90% was not associated with the increased prevalence of left carotid plaques in COPD patients. On the other hand, since 44.6% of the patients with COPD in our cohort presented both AHI ≥ 5 and ODI ≥ 10, there is an association between ODI and carotid plaque prevalence. However, AHI ≥ 5 included a higher number of patients than ODI ≥ 10, and also provides better fit of the model, suggesting that AHI is an independent predictor of atherosclerotic plaques in the left carotid artery. Nevertheless, longitudinal observational studies in larger cohorts of COPD patients with and without OS are needed to elucidate the precise mechanisms which could play a key role in the cardiovascular outcomes of these patients.

Distinct previous investigations have shown that ischemic cerebrovascular events correlate positively with the increase of cIMT measured by ultrasonography (26–28). More recently, a meta-analysis including 13,428 patients with asymptomatic non-stenotic carotid plaques (NSCP) reported that the presence of NSCP is more closely related to the risk of first-ever o recurrent ischemic stroke than is cIMT (29). One of the most striking finding of the present study is that the potential deleterious effects of OS on the progression of CAS in patients with COPD seem to be largely restricted to left carotid artery. Interestingly, in a population-based cohort study in which carotid MRI scanning was performed to 1,414 stroke-free participants, authors reported that carotid atherosclerotic plaque size and composition are not symmetrically distributed and that high-risk plaque features, such as intraplaque hemorrhage, are predominant in left-sided carotid plaques (30). Based on these previous reports and taken into account that we found COPD patients with OS presented a higher prevalence and larger atherosclerotic plaques in the left carotid artery than those without OS, we consider OS as a potentially-modifiable risk factor of CAS, and suggest that it might have potential implications on ischemic stroke risk, although this should be assessed with additional longitudinal studies designed for that purpose that were not initially in the scope of this research.

It is well known that patients with COPD are at increased risk of ischemic stroke compared to the general population (31, 32), but the mechanisms and molecular mediators underlying the stroke predisposition of COPD patients still remain to be defined. Patients with COPD have also higher risk for increased cIMT than healthy subjects. In this regard, Watanabe et al. (33) have recently demonstrated the association between cIMT and forced expiratory volume (FEV) below 70% and smoking experience. On the other hand, mechanisms underlying subclinical organ damage in the obstructive sleep apnea (OSA) setting are multifactorial, including endothelial dysfunction, hypertension, and vascular remodeling (also comprehending increased cIMT) (34). In the same line, studies by Altin et al. (35) and Wang et al. (36) described an association of cIMT with severe OSA and AHI. Conversely, Myśliński et al. (37) described absence of differences in early lesions between severe OSA patients and healthy controls. However, patients with COPD have increased vascular damage compared to healthy subjects. Thus, according to current evidence and the results of our study, we suggest that OS may impact on plaque development rather than early atherosclerosis onset. Besides traditional risk factors for ischemic stroke, such as aging, tobacco smoking, diabetes, or hypercholesterolemia and arterial hypertension, there is extensive evidence indicating that chronic low-grade systemic inflammation and oxidative stress, which are key pathophysiological drivers of both pathological wall remodeling in atherosclerosis (16, 38) and COPD (39–41), can also induce cerebrovascular dysfunction and structural alterations of cerebral vessels increasing the risk for ischemic stroke in COPD patients (42, 43). Regarding the impact of OS on cardiovascular morbidity and mortality in COPD patients, distinct large observational studies have yielded conflicting results (44–46). Our results shown herein favor the notion that OS should be considered as a risk factor for atherosclerosis in the left carotid artery, because we observed that COPD patients with an AHI ≥ 5 presented 4.5-fold higher risk for presence of atherosclerotic plaques in the left carotid artery, which were also larger in patients with OS. However, further prospective longitudinal case-control studies in large cohorts of well-characterized COPD patients are warranted in order to determine the real impact of OS on the incidence of major cardiovascular events such as ischemic stroke among others.

The major strength of the present study is the novelty of its design performing ultrasound IMT measurements of both carotid arteries to a large cohort of COPD patients and control subjects assessed by spirometry and cardiorespiratory polygraphy. However, this study design has some limitations because causal interpretations of the impact of OS in the risk of ischemic stroke cannot be drawn from a cross-sectional study and large longitudinal observational studies are needed to accomplish that endpoint. Moreover - although AHI ≥ 5 stays statistically significant as an independent predictor of left carotid plaque presence - our analysis shows very high confidence intervals, suggesting that our study cohort may be too small or heterogeneous and, hence, odds estimations should be interpreted with caution.

In conclusion, the present study provides the first evidence that the presence of OS in COPD patients is positively associated with lager left carotid atherosclerotic plaques, suggesting that ultrasound carotids assessment may be useful to identify those COPD patients at higher-risk for ischemic stroke to whom in-depth cerebrovascular evaluation should be recommended. Nevertheless, in order to prove the efficacy of this screening strategy, in terms of outcomes and cost-effectiveness, further longitudinal clinical studies in larger cohorts of COPD patients are warranted.

## Data Availability

The original contributions presented in the study are included in the article/**Supplementary Material**, further inquiries can be directed to the corresponding author/s.

## References

[1] DALYs, G.B.D. and H. Collaborators. Global, regional, and national disability-adjusted life-years (DALYs) for 359 diseases and injuries and healthy life expectancy (HALE) for 195 countries and territories, 1990–2017: a systematic analysis for the global burden of disease study 2017. Lancet. (2018) 392(10159):1859–922. 10.1016/S0140-6736(18)32335-3 30415748 PMC6252083

[2] Vestbo J Hurd SS Agustí AG Jones PW Vogelmeier C Anzueto A Global strategy for the diagnosis, management, and prevention of chronic obstructive pulmonary disease: GOLD executive summary. Am J Respir Crit Care Med. (2013) 187(4):347–65. 10.1164/rccm.201204-0596PP 22878278

[3] Soriano JB Zielinski J Price D. Screening for and early detection of chronic obstructive pulmonary disease. Lancet. (2009) 374(9691):721–32. 10.1016/S0140-6736(09)61290-3 19716965

[4] McNicholas WT. COPD-OSA Overlap syndrome: evolving evidence regarding epidemiology, clinical consequences, and management. Chest. (2017) 152(6):1318–26. 10.1016/j.chest.2017.04.160 28442310

[5] Shah AJ Quek E Alqahtani JS Hurst JR Mandal S. Cardiovascular outcomes in patients with COPD-OSA overlap syndrome: a systematic review and meta-analysis. Sleep Med Rev. (2022) 63:101627. 10.1016/j.smrv.2022.101627 35413500

[6] Shawon MS Perret JL Senaratna CV Lodge C Hamilton GS Dharmage SC. Current evidence on prevalence and clinical outcomes of co-morbid obstructive sleep apnea and chronic obstructive pulmonary disease: a systematic review. Sleep Med Rev. (2017) 32:58–68. 10.1016/j.smrv.2016.02.007 28169105

[7] Soler X Gaio E Powell FL Ramsdell JW Loredo JS Malhotra A High prevalence of obstructive sleep apnea in patients with moderate to severe chronic obstructive pulmonary disease. Ann Am Thorac Soc. (2015) 12(8):1219–25. 10.1513/AnnalsATS.201407-336OC 25871443 PMC5466175

[8] Ferrera MC Labaki WW Han MK. Advances in chronic obstructive pulmonary disease. Annu Rev Med. (2021) 72:119–34. 10.1146/annurev-med-080919-112707 33502902 PMC8011854

[9] Yin HL Yin SQ Lin QY Xu Y Xu HW Liu T. Prevalence of comorbidities in chronic obstructive pulmonary disease patients: a meta-analysis. Medicine. (2017) 96(19):e6836. 10.1097/MD.0000000000006836 28489768 PMC5428602

[10] Voulgaris A Archontogeorgis K Steiropoulos P Papanas N. Cardiovascular disease in patients with chronic obstructive pulmonary disease, obstructive sleep apnoea syndrome and overlap syndrome. Curr Vasc Pharmacol. (2021) 19(3):285–300. 10.2174/18756212MTA1hMzMj4 32188387

[11] Vanfleteren LE Spruit MA Groenen M Gaffron S van Empel VP Bruijnzeel PL Clusters of comorbidities based on validated objective measurements and systemic inflammation in patients with chronic obstructive pulmonary disease. Am J Respir Crit Care Med. (2013) 187(7):728–35. 10.1164/rccm.201209-1665OC 23392440

[12] Pinto LM Alghamdi M Benedetti A Zaihra T Landry T Bourbeau J. Derivation and validation of clinical phenotypes for COPD: a systematic review. Respir Res. (2015) 16:50. 10.1186/s12931-015-0208-4 25928208 PMC4460884

[13] Ji P Kou Q Zhang J. Study on relationship between carotid intima-media thickness and inflammatory factors in obstructive sleep apnea. Nat Sci Sleep. (2022) 14:2179–87. 10.2147/NSS.S389253 36540195 PMC9760046

[14] Trzepizur W Blanchard M Ganem T Balusson F Feuilloy M Girault JM Sleep apnea-specific hypoxic burden, symptom subtypes, and risk of cardiovascular events and all-cause mortality. Am J Respir Crit Care Med. (2022) 205(1):108–17. 10.1164/rccm.202105-1274OC 34648724

[15] Poh TY Mac Aogain M Chan AK Yii AC Yong VF Tiew PY Understanding COPD-overlap syndromes. Expert Rev Respir Med. (2017) 11(4):285–98. 10.1080/17476348.2017.1305895 28282995

[16] Back M Weber C Lutgens E. Regulation of atherosclerotic plaque inflammation. J Intern Med. (2015) 278(5):462–82. 10.1111/joim.12367 25823439

[17] Mortality, G.B.D. and C. Causes of Death. Global, regional, and national age-sex specific all-cause and cause-specific mortality for 240 causes of death, 1990–2013: a systematic analysis for the global burden of disease study 2013. Lancet. (2015) 385(9963):117–71. 10.1016/S0140-6736(14)61682-2 25530442 PMC4340604

[18] Libby P Ridker PM Hansson GK. Progress and challenges in translating the biology of atherosclerosis. Nature. (2011) 473(7347):317–25. 10.1038/nature10146 21593864

[19] Saba L Saam T Jager HR Yuan C Hatsukami TS Saloner D Imaging biomarkers of vulnerable carotid plaques for stroke risk prediction and their potential clinical implications. Lancet Neurol. (2019) 18(6):559–72. 10.1016/S1474-4422(19)30035-3 30954372

[20] Zhao YY Javaheri S Wang R Guo N Koo BB Stein JH Associations between sleep apnea and subclinical carotid atherosclerosis: the multi-ethnic study of atherosclerosis. Stroke. (2019) 50(12):3340–6. 10.1161/STROKEAHA.118.022184 31610764 PMC6878193

[21] Matthews DR Hosker JP Rudenski AS Naylor BA Treacher DF Turner RC. Homeostasis model assessment: insulin resistance and beta-cell function from fasting plasma glucose and insulin concentrations in man. Diabetologia. (1985) 28(7):412–9. 10.1007/BF00280883 3899825

[22] National Cholesterol Education Program Expert Panel on Detection, E. and A. Treatment of High Blood Cholesterol in. Third report of the National Cholesterol Education Program (NCEP) expert panel on detection, evaluation, and treatment of high blood cholesterol in adults (adult treatment panel III) final report. Circulation. (2002) 106(25):3143–421. 10.1161/circ.106.25.3143 12485966

[23] Landete P Fernandez-Garcia CE Aldave-Orzaiz B Hernandez-Olivo M Acosta-Gutierrez CM Zamora-Garcia E Increased oxygen desaturation time during sleep is a risk factor for NASH in patients with obstructive sleep apnea: a prospective cohort study. Front Med. (2022) 9:808417. 10.3389/fmed.2022.808417 PMC8906568 35280896

[24] Lloberes P Duran-Cantolla J Martinez-Garcia MA Marin JM Ferrer A Corral J Diagnosis and treatment of sleep apnea-hypopnea syndrome. Spanish society of pulmonology and thoracic surgery. Arch Bronconeumol. (2011) 47(3):143–56. 10.1016/j.arbres.2011.01.001 21398016

[25] Nambi V Chambless L Folsom AR He M Hu Y Mosley T Carotid intima-media thickness and presence or absence of plaque improves prediction of coronary heart disease risk: the ARIC (Atherosclerosis Risk In Communities) study. J Am Coll Cardiol. (2010) 55(15):1600–7. 10.1016/j.jacc.2009.11.075 20378078 PMC2862308

[26] O'Leary DH Polak JF Kronmal RA Manolio TA Burke GL Wolfson SK Jr. Carotid-artery intima and media thickness as a risk factor for myocardial infarction and stroke in older adults. Cardiovascular health study collaborative research group. N Engl J Med. (1999) 340(1):14–22. 10.1056/NEJM199901073400103 9878640

[27] Bots ML Hoes AW Hofman A Witteman JC Grobbee DE. Cross-sectionally assessed carotid intima-media thickness relates to long-term risk of stroke, coronary heart disease and death as estimated by available risk functions. J Intern Med. (1999) 245(3):269–76. 10.1046/j.1365-2796.1999.0442f.x 10205589

[28] Touboul PJ Elbaz A Koller C Lucas C Adrai V Chedru F Common carotid artery intima-media thickness and brain infarction: the etude du profil genetique de l’Infarctus cerebral (GENIC) case-control study. The GENIC investigators. Circulation. (2000) 102(3):313–8. 10.1161/01.CIR.102.3.313 10899095

[29] Singh N Marko M Ospel JM Goyal M Almekhlafi M. The risk of stroke and TIA in nonstenotic carotid plaques: a systematic review and meta-analysis. AJNR Am J Neuroradiol. (2020) 41(8):1453–9. 10.3174/ajnr.A6613 32646945 PMC7658878

[30] Selwaness M van den Bouwhuijsen Q van Onkelen RS Hofman A Franco OH van der Lugt A Atherosclerotic plaque in the left carotid artery is more vulnerable than in the right. Stroke. (2014) 45(11):3226–30. 10.1161/STROKEAHA.114.005202 25228259

[31] Sidney S Sorel M Quesenberry CP Jr DeLuise C Lanes S Eisner MD. COPD And incident cardiovascular disease hospitalizations and mortality: kaiser permanente medical care program. Chest. (2005) 128(4):2068–75. 10.1378/chest.128.4.2068 16236856

[32] Feary JR Rodrigues LC Smith CJ Hubbard RB Gibson JE. Prevalence of major comorbidities in subjects with COPD and incidence of myocardial infarction and stroke: a comprehensive analysis using data from primary care. Thorax. (2010) 65(11):956–62. 10.1136/thx.2009.128082 20871122

[33] Watanabe K Onoue A Omori H Kubota K Yoshida M Katoh T. Association between airflow limitation and carotid intima-media thickness in the Japanese population. Int J Chron Obstruct Pulmon Dis. (2021) 16:715–26. 10.2147/COPD.S291477 33776430 PMC7989542

[34] Cuspidi C Tadic M Gherbesi E Sala C Grassi G. Targeting subclinical organ damage in obstructive sleep apnea: a narrative review. J Hum Hypertens. (2021) 35(1):26–36. 10.1038/s41371-020-00397-0 32801297

[35] Altin R Ozdemir H Mahmutyazicioglu K Kart L Uzun L Ozer T Evaluation of carotid artery wall thickness with high-resolution sonography in obstructive sleep apnea syndrome. J Clin Ultrasound. (2005) 33(2):80–6. 10.1002/jcu.20093 15674835

[36] Wang S Cui H Zhu C Wu R Meng L Yu Q Obstructive sleep apnea causes impairment of the carotid artery in patients with hypertrophic obstructive cardiomyopathy. Respir Med. (2019) 150:107–12. 10.1016/j.rmed.2019.03.002 30961935

[37] Myslinski W Szwed M Szwed J Panasiuk L Brozyna-Tkaczyk K Borysowicz M Prevalence of target organ damage in hypertensive patients with coexisting obstructive sleep apnea. Ann Agric Environ Med. (2022) 29(2):294–9. 10.26444/aaem/149469 35767766

[38] Stocker R Keaney JF Jr. Role of oxidative modifications in atherosclerosis. Physiol Rev. (2004) 84(4):1381–478. 10.1152/physrev.00047.2003 15383655

[39] Rogliani P Ritondo BL Laitano R Chetta A Calzetta L Advances in understanding of mechanisms related to increased cardiovascular risk in COPD. Expert Rev Respir Med. (2021) 15(1):59–70. 10.1080/17476348.2021.1840982 33084434

[40] Brassington K Selemidis S Bozinovski S Vlahos R. Chronic obstructive pulmonary disease and atherosclerosis: common mechanisms and novel therapeutics. Clin Sci. (2022) 136(6):405–23. 10.1042/CS20210835 PMC8968302 35319068

[41] Balbirsingh V Mohammed AS Turner AM Newnham M. Cardiovascular disease in chronic obstructive pulmonary disease: a narrative review. Thorax. (2022). 10.1136/thoraxjnl-2021-218333 35772939

[42] Austin V Crack PJ Bozinovski S Miller AA Vlahos R. COPD And stroke: are systemic inflammation and oxidative stress the missing links? Clin Sci. (2016) 130(13):1039–50. 10.1042/CS20160043 PMC4876483 27215677

[43] Corlateanu A Covantev S Mathioudakis AG Botnaru V Cazzola M Siafakas N. Chronic obstructive pulmonary disease and stroke. J Chronic Obstr Pulm Dis. (2018) 15(4):405–13. 10.1080/15412555.2018.1464551 29746193

[44] Marin JM Soriano JB Carrizo SJ Boldova A Celli BR. Outcomes in patients with chronic obstructive pulmonary disease and obstructive sleep apnea: the overlap syndrome. Am J Respir Crit Care Med. (2010) 182(3):325–31. 10.1164/rccm.200912-1869OC 20378728

[45] Kendzerska T Leung RS Aaron SD Ayas N Sandoz JS Gershon AS. Cardiovascular outcomes and all-cause mortality in patients with obstructive sleep apnea and chronic obstructive pulmonary disease (overlap syndrome). Ann Am Thorac Soc. (2019) 16(1):71–81. 10.1513/AnnalsATS.201802-136OC 30372124

[46] Adler D Bailly S Benmerad M Joyeux-Faure M Jullian-Desayes I Soccal PM Clinical presentation and comorbidities of obstructive sleep apnea-COPD overlap syndrome. PLoS ONE. (2020) 15(7):e0235331. 10.1371/journal.pone.0235331 32645005 PMC7347183

