# Supplementary material for: Sleep apnea-COPD overlap syndrome is associated with larger left carotid atherosclerotic plaques
Source: Front Cardiovasc Med. 2023 Mar 21;10:1104377. doi: 10.3389/fcvm.2023.1104377 (PMC10070750; doi:10.3389/fcvm.2023.1104377)
Supplement: Supplementary file 1 [file Table1.docx]

**Supplementary** **Table 1. Pulmonary function parameter in study population**

| **Features** | **NLP**  **(n=32)** | **COPD**  **(n=74)** | ***p* value** |
| --- | --- | --- | --- |
| PRE FVC (ml) | 3680.94 ± 993.58 | 2627.18 ± 947.99 | **<0.001** |
| PRE FVC (%) | 96.87 ± 13.79 | 72.56 ± 19.83 | **<0.001** |
| PRE FEV (ml) | 2794.34 ± 1000.43 | 1423.15 ± 707.15 | **<0.001** |
| PRE FEV (%) | 101.53 ± 12.92 | 51.59 ± 20.20 | **<0.001** |
| PRE FEV/FVC | 80.19 ± 5.30 | 52.73 ± 13.11 | **<0.001** |
| POST FVC (ml) | 3680.31 ± 991.52 | 2707.54 ± 964.07 | **<0.001** |
| POST FVC (%) | 96.94 ± 14.02 | 74.63 ± 18.71 | **<0.001** |
| POST FEV (ml) | 2984.38 ± 788.73 | 1504.01 ± 751.93 | **<0.001** |
| POST FEV (%) | 102.84 ± 13.36 | 54.61 ± 21.71 | **<0.001** |
| POST FEV/FVC | 81.37 ± 5.42 | 53.42 ± 13.58 | **<0.001** |
| Basal saturation | 97.03 ± 1.20 | 93.57 ± 4.45 | **<0.001** |
| Minimum saturation | 84.24 ± 14.85 | 77.59 ± 9.76 | **<0.001** |
| Mallampati score | 1.13 ± 0.75 | 1.44 ± 0.89 | 0.103 |
| Dyspnoea grade | N/A | 1.78 ± 1.01 | N/A |
| Physical activity scale | N/A | 1.12 ± 0.76 | N/A |
| Exacerbations/year | N/A | 0.80 ± 1.11 | N/A |
| COPD score (Gold) | N/A | 1.39 ± 1.07 | N/A |

Data are shown as mean ± standard deviation or as number of cases (%). NLP, subjects with normal lung parameters; COPD, subjects with chronic obstructive pulmonary disease; FVC, forced vital capacity; FEV, forced expiratory volume; N/A, not aplicable.
