# Supplementary material for: Sleep apnea-COPD overlap syndrome is associated with larger left carotid atherosclerotic plaques
Source: Front Cardiovasc Med. 2023 Mar 21;10:1104377. doi: 10.3389/fcvm.2023.1104377 (PMC10070750; doi:10.3389/fcvm.2023.1104377)
Supplement: Supplementary file 2 [file Table2.docx]

**Supplementary** **Table 2. Characteristics of the COPD population according to OS presence**

| **Features** | **Non-OS**  **(n=36)** | **OS**  **(n=38)** | ***p* value** |
| --- | --- | --- | --- |
| Age (years) | 63.61 ± 6.63 | 63.16 ± 6.58 | 0.888 |
| Gender |  |  | **0.003** |
| Women, n (%) | 23 (63.9) | 11 (28.9) |  |
| Men, n (%) | 13 (36.1) | 27 (71.1) |  |
| Body mass index (kg/m^2^) | 26.32 ± 5.93 | 28.87 ± 5.46 | 0.063 |
| Body mass index ≥ 30, n (%) | 8 (22.2) | 16 (42.1) | 0.085 |
| Glucose (mg/dL) | 96.33 ± 21.03 | 107.05 ± 28.17 | 0.325 |
| Insulin levels (μU/L) | 13.47 ± 8.00 | 15.46 ± 10.97 | 0.495 |
| HOMA-IR score | 3.60 ± 2.53 | 4.26 ± 3.35 | 0.424 |
| HOMA-IR score ≥ 2.5, n (%) | 24 (66.7) | 26 (68.4) | 0.872 |
| Glycated Hb (%) | 5.62 ± 0.42 | 5.87 ± 0.69 | 0.150 |
| Type 2 diabetes mellitus, n (%) | 6 (16.7) | 9 (23.7) | 0.567 |
| Hypertension, n (%) |  |  |  |
| Dyslipidemia, n (%) | 16 (44.4) | 20 (52.6) | 0.481 |
| Metabolic syndrome, n (%) | 5 (13.9) | 7 (18.4) | 0.755 |
| Triglycerides (mg/dL) | 118.72 ± 63.39 | 131.00 ± 74.70 | 0.489 |
| Total cholesterol | 199.83 ± 39.46 | 196.71 ± 45.34 | 0.747 |
| HDL-cholesterol (mg/dL) | 64.31 ± 23.06 | 57.05 ± 21.06 | 0.184 |
| LDL cholesterol (mg/dL) | 106.56 ± 39.10 | 111.29 ± 38.63 | 0.636 |
| ALT (IU/L) | 21.36 ± 8.30 | 24.10 ± 9.71 | 0.248 |
| AST (IU/L) | 22.14 ± 7.67 | 24.16 ± 8.62 | 0.390 |
| GGT (IU/L) | 26.72 ± 18.04 | 45.87 ± 35.97 | **0.001** |
| Iron (μg/dL) | 87.89 ± 31.01 | 91.42 ± 27.95 | 0.360 |
| Ferritin (ng/mL) | 123.17 ± 86.93 | 144.76 ± 108.26 | 0.267 |
| Transferrin (mg/dL) | 257.92 ± 40.51 | 260.82 ± 38.95 | 0.983 |
| Alkaline phosphatase (IU/L) | 68.67 ± 17.74 | 73.66 ± 21.06 | 0.219 |
| Lactate dehydrogenase (U/L) | 191.97 ± 40.65 | 199.11 ± 41.27 | 0.484 |
| Albumin (g/dL) | 4.33 ± 0.31 | 4.43 ± 0.46 | 0.277 |
| Platelets (10^9^/L) | 0.24 ± 0.06 | 0.23 ± 0.05 | 0.744 |
| Total bilirubin (mg/dL) | 0.54 ± 0.28 | 0.56 ± 0.29 | 0.819 |
| C reactive protein (mg/L) | 0.37 ± 0.46 | 0.46 ± 0.57 | 0.459 |

Data are shown as mean ± standard deviation or as number of cases (%). NLP, subjects with normal lung parameters; COPD, subjects with chronic obstructive pulmonary disease; OS, subjects with overlap syndrome; HOMA-IR, homeostatic model assessment-insulin resistance; Hb, hemoglobin; HDL, high-density lipoprotein; LDL, low-density lipoprotein; VLDL, very low-density lipoprotein; ALT, alanine aminotransferase; AST, aspartate aminotransferase; GGT, gamma-glutamyltransferase.
