# Supplementary material for: Sleep apnea-COPD overlap syndrome is associated with larger left carotid atherosclerotic plaques
Source: Front Cardiovasc Med. 2023 Mar 21;10:1104377. doi: 10.3389/fcvm.2023.1104377 (PMC10070750; doi:10.3389/fcvm.2023.1104377)
Supplement: Supplementary file 3 [file Table3.docx]

**Supplementary** **Table 3. Univariate and multivariate logistic regression analysis of the independent variables associated with left carotid artery atherosclerotic plaque presence in COPD patients (n=74)**

| **Independent variables** | **Univariate analysis** | | | **Multivariate analysis** | | |
| --- | --- | --- | --- | --- | --- | --- |
|  | **OR** | **95% CI** | ***p* value** | **OR** | **95% CI** | ***p* value** |
| Age (years) | 1.11 | [1.03-1.21] | 0.011 | 1.19 | [1.07-1.32] | **0.002** |
| Sex (male/female) | 1.63 | [0.63-4.27] | 0.317 |  |  |  |
| BMI (kg/m^2^) | 1.05 | [0.96-1.14] | 0.283 |  |  |  |
| Diabetes (yes/no) | 0.54 | [0.17-1.72] | 0.299 |  |  |  |
| Hypertension (yes/no) | 1.63 | [0.63-4.27] | 0.317 |  |  |  |
| Total cholesterol (mg/dL) | 1.00 | [0.98-1.01] | 0.366 |  |  |  |
| Current smoking (yes/no) | 4.20 | [1.10-16.07] | 0.036 | 10.50 | [1.94-56.71] | **0.006** |
| POST FEV (%) | 1.00 | [0.98-1.02] | 0.934 |  |  |  |
| Exacerbations (number/year) | 0.99 | [0.64-1.52] | 0.952 |  |  |  |
| AHI ≥ 5 (events/hour) | 3.15 | [1.16-8.53] | 0.024 | 4.54 | [1.39-14.85] | **0.012** |
| Tc90% ≥ 10 (%) | 1.25 | [0.46-3.37] | 0.659 |  |  |  |
| Minimal O_2_ saturation (%) | 0.96 | [0.91-1.01] | 0.142 |  |  |  |

OR, odds ratio; CI, confidence interval; BMI, body mass index; FEV, forced expiratory volume; AHI, apnea-hypopnea index: Tc90%, time with oxygen saturation below 90% during sleep.
